# Regulatory T Cell Metabolism in Atherosclerosis

**DOI:** 10.3390/metabo10070279

**Published:** 2020-07-08

**Authors:** Jeroen Baardman, Esther Lutgens

**Affiliations:** 1 Department of Medical Biochemistry, Experimental Vascular Biology, Amsterdam Cardiovascular Sciences, Amsterdam UMC, University of Amsterdam, 1105 AZ Amsterdam, The Netherlands; e.lutgens@amsterdamumc.nl; 2 Institute for Cardiovascular Prevention (IPEK), Klinikum der Universität München (KUM), Ludwig-Maximilians-Universität (LMU) München, 80336 Munich, Germany; 3 German Center for Cardiovascular Research (DZHK), Partner Site Munich Heart Alliance, 80336 Munich, Germany

**Keywords:** Tregs, Foxp3, T cells, metabolism, immunometabolism, atherosclerosis, hypercholesterolemia

## Abstract

Regulatory T cells (Tregs) are capable of suppressing excessive immune responses to prevent autoimmunity and chronic inflammation. Decreased numbers of Tregs and impaired suppressive function are associated with the progression of atherosclerosis, a chronic inflammatory disease of the arterial wall and the leading cause of cardiovascular disease. Therefore, therapeutic strategies to improve Treg number or function could be beneficial to preventing atherosclerotic disease development. A growing body of evidence shows that intracellular metabolism of Tregs is a key regulator of their proliferation, suppressive function, and stability. Here we evaluate the role of Tregs in atherosclerosis, their metabolic regulation, and the links between their metabolism and atherosclerosis.

## 1. Introduction

Atherosclerosis is a chronic lipid-driven inflammatory disease of the arterial vasculature and the main underlying pathological cause of cardiovascular disease. Subendothelial accumulation of cholesterol-rich lipoproteins, particularly low-density-lipoprotein (LDL), at susceptible sites of the arterial wall, triggers the infiltration of leukocytes. In the vascular intima, LDL particles may undergo modification to oxidative (ox) LDL which leads to the activation of vascular and innate immune cells. Additionally, LDL-derived peptides serve as antigens that drive T cell activation in atherosclerosis and further aggravate inflammatory responses. Ongoing innate and adaptive immune responses promote plaque progression and eventual rupture of unstable plaques that may lead to thrombus formation and acute clinical manifestations such as myocardial infarction and stroke.

Increasing evidence emphasizes the role of T cells as important drivers and modulators of atherogenesis [1,2]. Regulatory T cells (Tregs) are capable of suppressing exacerbated inflammatory responses to enforce immunological tolerance and homeostasis [3,4]. This specialized subset of CD4^+^ T cells is phenotypically characterized by constitutively high expression of the interleukin (IL)-2 receptor α chain (CD25) on their surfaces. In addition, transcription factor forkhead box protein 3 (Foxp3) serves as lineage-specific marker and master regulator for Tregs. Most of the Foxp3^+^ Tregs are generated in the thymus and are termed natural Tregs (nTregs), while Tregs may also differentiate from conventional T cells at peripheral sites or in vitro as induced Tregs (iTregs). Tregs suppress inflammatory responses via multiple mechanisms, including the suppression of effector T cell proliferation; secretion of immunomodulatory cytokines, such as IL-10 and transforming growth factor beta (TGF-β); and inhibition of antigen presentation by antigen-presenting cells (APCs) via co-inhibitory molecules such as cytotoxic T-lymphocyte-associated protein 4 (CTLA-4). Depletion of CD4^+^CD25^+^ T cells gives rise to a broad-spectrum of autoimmune diseases in mice, whereas reconstitution of CD4^+^CD25^+^ T cells prevents autoimmune disease. In humans, loss-of-function mutations of *FOXP3* induces immunodysregulation polyendocrinopathy enteropathy X-linked (IPEX) syndrome, a syndrome characterized by severe autoimmunity due to dysfunctional Tregs.

Numerous studies show that deficiency or dysfunction of Tregs is also associated with the development of atherosclerosis [5]. Therefore, strategies to increase their number or improve their suppressive activity are potential tools to combat atherogenesis.

As is true for all immune cells, cellular metabolism is the key driver of Treg function [6,7]. Targeting intracellular metabolic pathways in immune cells has arisen as a promising approach to prevent atherosclerotic disease development [8]. In this review we will discuss the role of Tregs in atherosclerosis, their metabolic regulation, and the putative links between their cellular metabolism and atherogenesis.

## 2. Treg Numbers in Atherosclerosis

A large body of evidence from experimental and clinical studies shows that decreased numbers of Tregs are associated with the development of atherosclerosis [5]. Reduced Treg numbers were observed in the lymphoid organs of atherosclerotic apolipoprotein E-deficient (*Apoe*^−/−^) mice, compared to wild type littermates or young *Apoe*^−/−^ mice with no detectable atherosclerotic plaques [9]. In another murine atherosclerosis model using LDL receptor-deficient (*Lldr*^−/−^) mice, diet-induced hypercholesterolemia initially resulted in an increase of circulating and plaque Tregs, but their numbers were found to decline in later stages of the disease [10]. In humans, Tregs are scarce in atherosclerotic plaques during all stages of development [11]. However, several clinical studies demonstrate that the numbers of circulating Tregs are reduced in patients with acute coronary syndrome (ACS) compared to individuals with stable angina or normal coronary arteries [12,13,14,15]. Accordingly, a large prospective cohort study demonstrated an association between low Treg frequencies and increased risk for developing acute coronary events [16]. Elimination of Tregs in *Apoe*^−/−^ mice using a CD25-specific antibody resulted in larger atherosclerotic lesions with more macrophages and T cells, and less collagen, indicative of enhanced plaque instability [17]. Similarly, irradiated *Ldlr*^−/−^ mice that received bone marrow from DEREG (depletion of regulatory T cells) mice, in which Tregs are specifically depleted, developed more severe diet-induced atherosclerosis [18]. Accordingly, adoptive transfer of Tregs into *Apoe*^−/−^ mice hampered atherosclerotic development [9,17,19].

Whereas Treg numbers progressively decrease in aortas of *Ldlr*^−/−^ mice during disease development, effector T cells accumulate in these plaques [10]. Within human atherosclerotic plaques, T helper 1 (Th1) cells are the most prevalent subset of T cells [20]. Th1 cells are characterized by their expression of the lineage-defining transcription factor T-bet and produce high levels of interferon-γ (IFNγ). This cytokine enhances plaque progression and destabilization via multiple mechanisms, including promoting leukocyte recruitment to atherosclerotic plaques, foam cell formation, and inhibition of vascular smooth muscle cell proliferation and their production of collagen [21]. Likewise, *Ldlr*^−/−^ mice deficient for T-bet or IFNγ developed less atherosclerosis [22,23]. Altogether, these findings indicate that an imbalance between proatherogenic Th1 cells and atheroprotective Tregs during atherosclerosis drives disease progression. Additionally, other T cell subsets, including Th2 and Th17 cells, may also dictate atherosclerotic disease development [2]. However, the exact role of Th2 and Th17 cells during atherogenesis remains controversial, since both proatherogenic and atheroprotective activities have been linked to these T cell subsets.

## 3. Atheroprotective Functions of Tregs

In addition to reduced Treg numbers, multiple studies reported an impaired suppressive function of Tregs during atherosclerosis [5]. Tregs exploit multiple mechanisms to suppress inflammatory responses, including the secretion of inhibitory cytokines IL-10 and TGF-β [24]. Deficiency of IL-10 in mice results in the formation of larger atherosclerotic plaques with reduced collagen content [25]. Moreover, there was increased infiltration of T cells into the plaques of IL-10 deficient mice, accompanied by elevated levels of proatherogenic IFNγ. In contrast, overexpression of IL-10 in activated T cells reduces atherosclerosis in *Ldlr*^−/−^ mice and was found to decrease IFNγ production in circulating lymphocytes and monocytes [26]. In support of these findings, lower levels of circulating IL-10 were detected in patients with ACS [15]. Likewise, abrogation of TGF-β signaling in *Apoe*^−/−^ mice was found to result in more advanced atherosclerosis while TGF-β overexpression reduces atherosclerosis [27,28,29].

It has been increasingly recognized that T cells drive autoimmune responses in atherosclerotic plaques against atherosclerosis-specific antigens [2]. Compelling evidence indicates that apolipoprotein B (ApoB), the core protein of LDL, serves as such an atherosclerosis-specific autoantigen. ApoB-specific CD4^+^ T cells are detected in human blood and Apoe^−/−^ mice [30]. Interestingly, in healthy donors most of these CD4^+^ T cells are Foxp3^+^ Tregs, whereas Tregs of donors with subclinical cardiovascular disease co-express T-bet or the Th17 lineage transcription factor receptor retinoic acid receptor-related-orphan-receptor-gamma t (RORγt). Autoantigens in atherosclerosis are presented to T cells via major histocompatibility complex (MHC) class II on APCs. In addition to recognition of this MHC-antigen complex on APCs, T cells require a secondary signal for their activation. This second signal is provided by binding of CD28 on T cells with co-stimulatory molecules such as CD80 and CD86 on the surface of APCs. Dendritic cells (DCs) are the most important APCs and therefore essential cells in eliciting proatherogenic T cell-mediated responses. Tregs can suppress DC-induced T cell activation via the expression of co-inhibitory molecules such as CTLA-4 on their surface. CTLA-4 binds to CD80 and CD86 on DCs, thereby preventing the interaction of CD80/CD86 with CD28 and disrupting T cell activation. In addition, to compete with CD28 for CD80/CD86 binding, CTLA-4 also eliminates CD80/CD86 surface expression on APCs via transendocytosis. Overexpression of CTLA-4 in *Apoe*^−/−^ mice was found to reduce CD80/CD86 expression on DCs, accompanied by suppressed T cell activation and reduced atherosclerosis [31].

Tregs also possess the capability to modulate macrophage responses in the context of atherogenesis. Murine Tregs were found to downregulate scavenger receptors on macrophages, thereby blunting their capacity to take up cholesterol, resulting in suppressed foam cell formation [32]. Transformation of macrophages into lipid-loaded foam cells is a critical event in the development of atherosclerosis [33]. Deregulated cholesterol homeostasis within macrophage foam cells can lead to apoptosis. In early stages of atherosclerosis, such dying macrophages are efficiently cleared by other macrophages via efferocytosis. However, in more advanced stages, defective efferocytosis and increased macrophage apoptosis can lead to secondary necrosis and the formation of a necrotic core, associated with unstable plaques. Recently, it was shown that Tregs enhance macrophage efferocytosis via their secretion of IL-13 [34]. Treg-derived IL-13 stimulates the expression of IL-10 in macrophages and this optimizes their capacity to internalize apoptotic cells. Additionally, macrophages may accelerate atherosclerotic plaque progression via the secretion of inflammatory cytokines, chemokines, and proteases. Both human and murine Tregs were found to inhibit pro-inflammatory responses of macrophages and skew them towards an anti-inflammatory phenotype [32,35].

## 4. Cellular Metabolism

**Figure 1:**
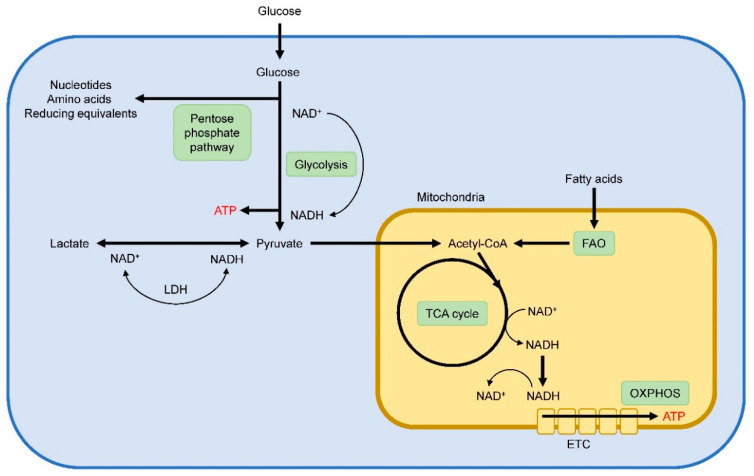
Schematic overview of main metabolic pathways within cells.

In order to meet their energetic demands for survival and function, cells exploit distinct metabolic programs to meet their energy and biosynthetic demands. Within mammalian cells, glycolysis and oxidative phosphorylation (OXPHOS) are two major metabolic pathways to deliver ATP (Figure 1). During glycolysis, extracellular glucose is imported and enzymatically broken down to pyruvate via a multistep process in the cytosol. Subsequently, pyruvate can either be converted to lactate or transported into the mitochondria to fed OXPHOS. To undergo oxidative metabolism, pyruvate is converted to acetyl-CoA in the mitochondria, which serves as a substrate for the TCA cycle. In the TCA cycle, essential electronic donors are generated, which can fuel the electronic transport chain (ETC) to drive ATP generation via OXPHOS. In addition, breakdown of fatty acids to acetyl-CoA via fatty-acid β-oxidation (FAO) is also used to feed the TCA cycle for subsequent ATP production via OXPHOS. For OXPHOS, oxygen must be present, and in oxygen limited conditions pyruvate will be metabolized to lactate and will not undergo oxidative metabolism. During glycolysis, nicotinamide adenine dinucleotide (NAD^+^) is reduced to form NADH, and cells need to regenerate NAD^+^ in order to continue glycolysis. In oxygen-sufficient conditions, NADH is oxidized to NAD^+^ in the mitochondria. However, conversion of pyruvate into lactate trough lactate dehydrogenase (LDH) enables the NAD^+^ generation to maintain a continued glycolytic flux under conditions of oxygen depletion. Compared to OXPHOS, glycolysis is an inefficient energy producing pathway. Despite the presence of abundant oxygen and functional mitochondria, cells may metabolize most of their glucose into lactate and employ glycolysis as their major source for energy supply. This phenomenon is known as aerobic glycolysis, or the Warburg effect, and is typical for rapidly proliferating cells. Increased glycolytic flux facilitates the diversion of glycolytic intermediates into biosynthetic pathways such as the pentose phosphate pathway to generate nucleotides, amino acids, and reducing equivalents for anabolic cell growth and proliferation.

## 5. Treg Metabolism

It is now increasingly recognized that cellular metabolism also dictates T cell differentiation and function [36,37,38]. Resting T cells exhibit a catabolic metabolism and predominantly rely on FAO and the oxidation of glucose-derived pyruvate through the TCA cycle. Following T cell receptor (TCR) stimulation, T cells undergo a metabolic shift toward anabolic metabolism to support rapid cell growth and proliferation. Initial experiments with in vitro differentiated T cells from mice demonstrated that effector T cells are highly glycolytic while iTregs predominantly rely on FAO [39]. Compared with effector T cells, iTregs have lower expression of the glucose transporter 1 (Glut 1) and exhibit lower rates of glucose import and glycolysis. Conversely, iTregs show increased rates of lipid oxidation and both nTregs and iTregs display strongly induced levels of the AMP-activated protein kinase (AMPK). As a critical regulator of metabolism, AMPK signaling limits anabolic cell growth by stimulating ATP-generating catabolic pathways and inhibiting ATP-consuming anabolic pathways [40]. For instance, AMPK inhibits the activity of acetyl-CoA carboxylase (ACC), a crucial enzyme for fatty acid synthesis. ACC catalyzes the conversion of acetyl-CoA into malonyl-CoA, the first committed step in the synthesis of fatty acids. Moreover, malonyl-CoA serves as an allosteric inhibitor of carnitine palmitoyltransferase I (CPT1), the rate-limiting enzyme in FAO. AMPK activator 5-aminoimidazole-4-carboxamide ribonucleotide (AICAR) was found to stimulate the uptake of fatty acids and mitochondrial biogenesis and enhance murine CD4^+^Foxp3^+^ Treg differentiation in vivo and in vitro [41]. Accordingly, pharmacological blocking of FAO with CPT1-inhibitor etomoxir suppressed the formation of murine iTregs [39,41]. Therefore, these results indicate that AMPK-driven FAO is crucial for the differentiation into iTregs. However, a genetic approach challenged this idea recently and demonstrates that FAO is largely dispensable for murine iTreg generation [42]. Etomoxir-induced inhibition of Treg development and function was found to be independent of CPT1 and suggested to be consequence of off-target effects that impair mitochondrial respiration. At high concentrations, etomoxir reduces OXPHOS in iTregs. Similarly, high concentrations of etomoxir were found to impair OXPHOS in macrophages, with off-target effects including the inhibition of ETC complex I [43]. Interestingly, Tregs from mice carrying a mitochondrial DNA mutation that impairs ETC complex I display reduced suppressive function [44]. In addition to genetic targeting, pharmacological inhibition of ETC complex I with rotenone impairs suppressive function of murine iTregs [45]. Recently, Treg-specific deletion of ETC complex III in mice was found to result in blunted suppressive Treg function and the development of a fatal inflammatory disease [46]. Altogether, these findings clearly indicate that mitochondrial respiration is essential for Tregs to maintain their suppressive function.

Another central coordinator of T cell metabolism is the evolutionarily conserved serine/threonine protein kinase mammalian target of rapamycin (mTOR), which consists of two multiprotein complexes [47]. mTOR complex 1 (mTORC1) signaling promotes anabolic processes for cellular growth, including the induction of Glut1 expression and aerobic glycolysis. Foxp3 negatively regulates mTORC1 signaling, aerobic glycolysis and proliferation while it promotes oxidative metabolism [44,48]. Mechanisms by which Foxp3 suppress glycolysis include inhibition of Glut1 and Myc, an important transcription factor for upregulating glycolysis. Blocking glycolysis with 2-deoxyglucose or mTOR using rapamycin has been shown to promote Foxp3 expression [49]. Conversely, Treg-specific deletion of tuberous sclerosis complex 1 (TSC1) in mice, a negative regulator of mTOR, induces constitutive activation of mTORC1 signaling and abrogates Foxp3 expression and suppressive Treg function [50].

**Figure 2:**
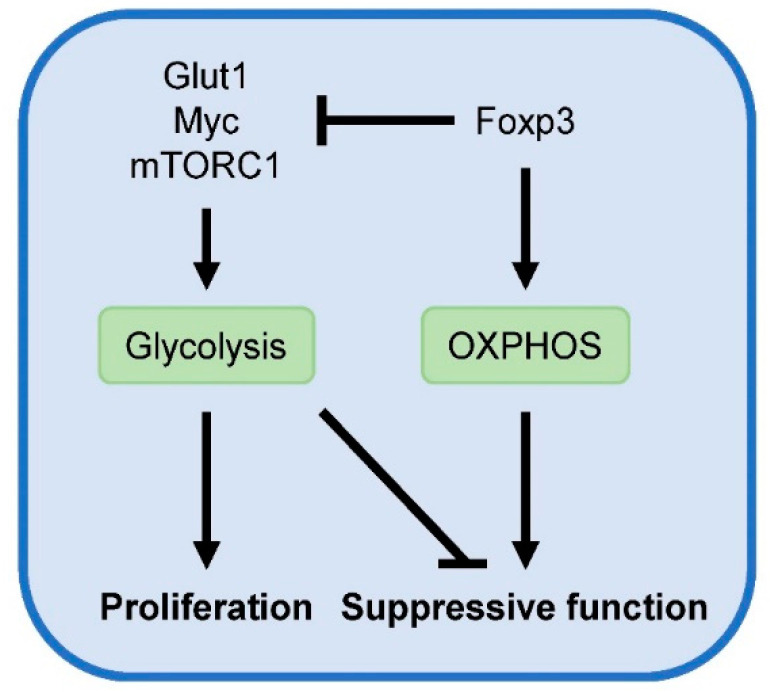
Metabolic regulation of Treg activity. Glycolysis fuels Treg proliferation and is associated with reduced suppressive function. Conversely, Foxp3 suppresses glycolysis and promotes OXPHOS, required for suppressive Treg activity.

Although chronic overactivation of mTOR signaling is detrimental for Foxp3 expression and Treg function, Treg-specific deletion of mTORC1 in mice results in a critical loss of Treg suppressive activity and the development of a fatal early-onset inflammatory disorder [51]. Additionally, mTORC1 and glucose metabolism were found to be essential for the suppressive activities of human nTregs [52]. Compared to human effector T cell subsets, human nTregs exhibit higher levels of glycolytic genes and metabolites, lactate production, and glucose uptake. This heightened glucose consumption by Tregs deprives glucose from responder T cells, resulting in DNA damage and senescence in these cells. Analysis of proliferating and non-proliferating nTregs from the spleens of wild type mice shows that Glut1 expression and mTORC1 activity are strongly elevated in proliferating Tregs [48]. Moreover, elevated glucose uptake and glycolytic activity upon transgenic expression of Glut1 is accompanied by increased Treg number and size. In agreement, freshly isolated human Tregs are highly proliferative and demonstrate a proteomic and metabolic signature towards elevated glycolysis [53]. Additionally, both glycolysis and FAO are required for human Treg proliferation in vitro. However, while transgenic expression of Glut1 promotes murine iTreg proliferation, it blunts their suppressive activity [48]. Similarly, TSC1-deficient Tregs with overactive mTORC1 activity are highly proliferative but exhibit compromised suppressive function [50]. These findings indicate that mTORC1 and aerobic glycolysis promote Treg proliferation at the cost of suppressive activity (Figure 2). Accordingly, oscillatory switches of mTOR activity are proposed to maintain optimal Treg homeostasis [54].

TCR stimulation strength is a crucial determinant for mTOR signaling and T cell differentiation [55]. Whereas strong TCR signaling induces T helper cell differentiation, low TCR signal strength and mTOR signaling promote the induction of Treg cells. For their in vitro differentiation towards Tregs, naïve T cells are commonly cultured in the presence of high concentrations of TGF-β and strong TCR stimulation. The addition of TGF-β (which was recently found to exhibit the intrinsic capacity to suppress glycolysis in murine Tregs via mTOR inhibition [56]) allows the generation of iTregs after strong TCR stimulation, since it mimics weak TCR activation [57]. Weak stimulation of the TCR was found to induce the generation of human iTregs, even in the absence of TGF-β [58]. This approach revealed that glycolysis is indispensable for the conversion of conventional T cells into Tregs, via the activity of enolase-1. This glycolytic enzyme also possesses non-glycolytic (moonlighting) activity and acts as a repressor of *FOXP3*. In resting conventional T cells, enolase-1 is bound to the promotor and regulatory regions of *FOXP3* in the nucleus to repress its expression. Weak TCR activation leads to the induction of glycolysis in T cells and translocation of enolase-1 from the nucleus to the cytosol to support glycolysis, thereby losing its capacity to repress *FOXP3* expression.

Altogether, these results indicate that distinct intracellular metabolic pathways may be exploited during different stages of Treg development [7]. Initially, weak TCR engagement activity in naïve T cells induces a metabolic shift from oxidative metabolism to glycolysis, leading to induction of Foxp3 transcription. Subsequently, Foxp3 transcription promotes oxidative metabolism to stabilize suppressive Treg function.

## 6. Hypercholesterolemia and Treg Stability and Suppressive Function

Since Tregs are highly sensitive to metabolic changes [57], specific systemic and local metabolic environmental signals during the development of atherosclerosis may negatively affect their atheroprotective functions. High levels of plasma cholesterol cause atherosclerosis [59] and are known to affect Treg function. For instance, Tregs isolated from hypercholesterolemic *Apoe*^−/−^ mice exhibit an attenuated capability to suppress the proliferation of effector T cells compared to their wild type littermates [9]. Hypercholesterolemia-induced disturbances of intracellular cholesterol metabolism may underlie this dysfunctional Treg phenotype.

Emerging evidence highlights the essential role of cholesterol metabolism in maintaining Treg function. For example, cholesterol metabolism was the most deregulated pathway when comparing wild type and mTORC1 deficient Tregs [51]. Loss of mTORC1 signaling in Tregs results in the downregulation of many genes involved in cholesterol biosynthesis, including that encoding 3-hydroxy-3-methylglutaryl-CoA reductase (HMGCR), the rate-limiting enzyme of the mevalonate pathway. This pathway produces isoprenoids such as cholesterol and geranylgeranyl pyrophosphate (GGPP) that are essential for many cellular processes. Statin-induced inhibition of HMGCR diminished the suppressive activity of Tregs and was found to reduce the expression of suppressive effector molecules CTLA-4 and ICOS on Tregs, thereby providing a mechanistic explanation for the development of profound inflammatory diseases in mice with mTORC1-deficient Tregs.

Recently, the importance of the mevalonate pathway for proper Treg function was further emphasized in Treg-specific liver kinase B1 (LKB1)-deficient mice [60]. LKB1 is a serine-threonine kinase that directly activates AMPK. Multiple groups have demonstrated that Treg-specific deletion of LKB1 in mice leads to the development of fatal autoimmune disease [60,61,62,63]. Surprisingly, genetic ablation of AMPK in Tregs did not result in any abnormalities in mice, indicating that the regulatory function of LKB1 in Tregs is independent of AMPK. Loss of LKB1 diminished expression of essential mevalonate pathway genes, including those encoding hydroxymethylglutaryl-CoA synthase (HMGCS) and HMGCR, indicating that LKB1 activity is crucial for the activation of this pathway. LKB1-deficient Tregs exhibit impaired suppressive activity, and this could be restored with the addition of mevalonate, an intermediate of the mevalonate pathway. Moreover, mevalonate-treated Treg-specific deficient LKB1 mice exhibited decreased autoimmune inflammation and increased survival.

Interestingly, a substantial fraction of the LKB1-deficient Tregs starts to produce cytokines that are associated with effector T cell subsets, including the inflammatory Th1 cytokine IFNγ [60]. It has been observed that under inflammatory conditions, Tregs can adopt phenotypical characteristics of effector T cells [64]. Such Tregs may acquire an effector-like phenotype and maintain Foxp3 expression (plasticity) or lose Foxp3 expression and become an ex-Treg cell (instability). During experimental murine atherosclerosis, it was found that Tregs can undergo plasticity and adapt an effector Th1-like phenotype, resulting in the accumulation of IFNγ secreting Treg/Th1 hybrid cells in plaques [65]. These plastic Th1/Treg cells maintain Foxp3 expression but exhibit no suppressive activity. A crucial regulator of Treg suppressive function is miR-146a, which downregulates the key Th1 transcription factor transducer and activator transcription 1 (STAT1) and prevents the conversion of Tregs in IFNγ-producing Th1-like cells [66]. Adoptive transfer of plasticity-prone *Mir146a*^−/−^ Tregs cells fails to reduce atherosclerosis in *Apoe*^−/−^ recipient mice [65]. Tregs may also completely lose their Foxp3 expression during atherosclerosis and are then converted into proatherogenic T follicular helper (Tfh) cells [67]. Multiple lines of evidence demonstrate that inhibition of the mevalonate pathway promotes the conversion of Tregs into effector-like T cells. For instance, purified Tregs from statin-treated mice produce high levels of IFNγ [60]. Similarly, short hairpin RNA-mediated knockdown of HMGCS1 in murine Tregs leads to the increased production of IFNγ. Conversely, treatment with mevalonate or GGPP suppresses the abnormal production of IFNγ in LKB1-deficient Tregs and improves their impaired suppressive activity. Moreover, mevalonate and GGPP restored the levels of CD25^+^Foxp3^+^ Tregs in the spleen of Treg-specific deficient LKB1 mice. GGPP was found to enhance IL-2 induced phosphorylation of STAT5, a transcription factor that activates and sustains Fox3 expression. GGPP was found to enhance IL-2 induced phosphorylation of STAT5, known to activate and sustain Foxp3. Thus, these findings indicate that activation of the mevalonate pathway is indispensable for the maintenance of Treg stability and function.

Surprisingly, whereas the levels of mevalonate and GGPP were reduced in LKB1-deficient Tregs, the total amount of cholesterol was increased in these cells [60]. In contrast to treatment with mevalonate and GGPP, cholesterol failed to restore CD25^+^Foxp3^+^ frequencies in Treg-specific deficient LKB1 mice. Moreover, treatment of wild type Tregs with cholesterol promotes the production of IFNγ in these cells. These results indicate that accumulation of cholesterol within Tregs negatively affect their functional integrity. Balanced influx and efflux of cholesterol is critical to maintaining cholesterol homeostasis within cells. Uptake of cholesterol by cells is primarily mediated trough the LDL receptor and the scavenger receptor CD36, whereas the lipid transporters ATP-binding cassette transporter A1 (ABCA1) and G1 (ABCG1) are essential for cholesterol efflux. Maintenance of intracellular cholesterol homeostasis is coordinated through the opposing transcriptional activities of sterol regulatory element binding protein 2 (SREBP-2) and the liver X receptor (LXR). When levels of cellular cholesterol are low, SREBP-2 is activated to induce the expression of genes involved in cholesterol uptake and cholesterol biosynthesis. Alternatively, excessive cellular cholesterol leads to the activation of LXR which promotes cholesterol efflux and limits the uptake of cholesterol. Accordingly, treatment of wild type murine Tregs with cholesterol suppresses the expression of mevalonate pathway genes in these cells [60]. Interestingly, LKB1-deficient Tregs exhibit increased expression of *Cd36* and reduced expression of *Abca1* and *Abcg1*, indicating that LKB1 is an important regulator for cholesterol homeostasis in Tregs. Thus, accumulation of cholesterol decreases the demand for cholesterol, resulting in suppressed activation of the mevalonate pathway, which is not only essential for cholesterol biosynthesis but also generates metabolites that are critical for maintenance of Treg stability and function.

The crucial relationship between intracellular cholesterol homeostasis and Treg stability has also been emphasized in experimental murine atherosclerosis [67]. Elevated plasma cholesterol in western diet-fed *Apoe*^−/−^ mice was accompanied by an increase of esterified cholesterol in Tregs from these mice. This demonstrates that hypercholesterolemia disrupts intracellular cholesterol homeostasis, leading to the accumulation of cholesterol in Tregs. Treatment of western diet-fed *Apoe*^−/−^ mice with apolipoprotein AI (ApoAI) increases the expression of cholesterol efflux transporter ABCA1 in Tregs and normalizes cholesterol content in these cells. Of interest, ApoA1 treatment was found to inhibit the conversion of Tregs into proatherogenic Tfh cells and reduces atherosclerosis in western diet-fed *Apoe*^−/−^ mice. Suppressed IL-2 signaling (crucial for Foxp3 expression and Treg maintenance) and increased IL-6Rα expression (important of Tfh differentiation) were proposed to drive the conversion of Tregs into Tfh cells during experimental murine atherosclerosis. Mechanistically, accumulation of intracellular cholesterol was hypothesized to affect membrane lipid rafts in which IL-2 receptors are enriched, thereby abolishing IL-2 signaling and Treg function and homeostasis.

**Figure 3:**
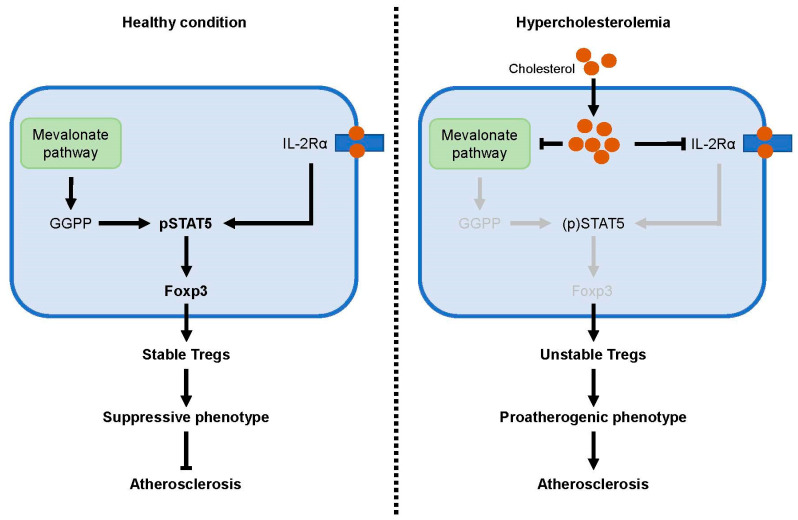
Hypercholesterolemia disrupts intracellular cholesterol homeostasis and Treg stability. IL-2 signaling induces phosphorylation of STAT5, known to induce and sustain Foxp3 expression. GGPP, a product of the mevalonate pathway, is indispensable for Treg stability and enhances STAT5 phosphorylation. Accumulation of intracellular cholesterol during hypercholesterolemia may inhibit the mevalonate pathway or reduce expression of IL-2Rα, leading to diminished Treg stability. Unstable Tregs can lose their suppressive phenotype and adapt proatherogenic features, thereby promoting atherosclerotic disease development.

Collectively, these experimental findings indicate that intracellular cholesterol homeostasis is crucial for maintenance of Treg stability and function. Massive accumulation of cellular cholesterol, such as during hypercholesterolemia, may disrupt this homeostasis and promote disease progression via the conversion of Tregs into proatherogenic effector T cells (Figure 3).

## 7. Hypercholesterolemia and Treg Migration

Impaired migratory ability is proposed as a possible mechanism for the decreased Treg numbers in atherosclerotic lesions after prolonged hypercholesterolemia in mice [10]. Whereas hypercholesterolemia initially induces an accumulation of Tregs in the atherosclerotic aorta of *Ldlr*^−/−^ mice, their numbers decrease over time. A concomitant increase of Tregs was observed in the spleens of these mice, supporting the hypothesis of diminished Treg migration to atherosclerotic lesions during hypercholesterolemia. Motility is the amongst the most energy-consuming cellular activities, and it was found that glucokinase (GCK)-mediated glycolysis drives Treg migration [68]. GCK is induced in a mTORC2-mediated manner and interacts with actin to supply ATP-hydrolyzing sodium pump (Na, K-ATPase) with glycolytic-ATP and promote cytoskeletal arrangements. Such actin remodeling enables Treg cells to squeeze through capillaries. Both mTORC2-depleted and GCK-depleted Tregs display a severely impaired capacity to migrate into inflammatory sites. Engagement of the co-stimulatory molecule CD28 or lymphocyte function-associated antigen (LFA-1) integrin, a key regulator of T cell migration, was found to induce glycolysis and GCK expression in Tregs. Moreover, stimulation of either CD28 or LFA-1 downregulates the expression of the GCK regulatory protein, a competitive inhibitor of GCK. Interestingly, expression of LFA-1 on splenic Tregs from *Ldlr*^−/−^ mice was strongly reduced during hypercholesterolemia [10]. Therefore, these data imply that hypercholesterolemia affects motility-associated glycolysis and GCK activity in Tregs. However, additional research is needed to determine the influence of hypercholesterolemia on GSK-induced glycolysis and Treg migration.

## 8. Atherosclerotic Plaque Microenvironment and Treg Function

Targeted metabolomic analysis of human carotid atherosclerotic plaques identified an altered metabolic profile in high-risk plaques that associated with cerebrovascular symptoms in patients and histologically assessed vulnerability compared with stable low-risk plaques [69]. High-risk plaques show reduced concentrations of hexoses (predominately glucose) and elevated levels of lactate, indicative of increased aerobic glycolysis. Interestingly, Foxp3-induced metabolic reprogramming enables Tregs to withstand low glucose-high lactate environments [44]. Exposure to lactate impairs effector T cell proliferation, while proliferation and suppressive function of Treg remains unaffected. Influx of high levels of lactate and subsequent oxidation of lactate to pyruvate via LDH in T cells blunt their capability to generate NAD^+^, required to maintain glycolysis. This is especially deleterious for effector T cell function, which depends on glycolysis for the production of inflammatory mediators. Foxp3 suppresses glycolysis (NAD^+^ consuming pathway) and promotes OXPHOS (NAD^+^ producing pathway), resulting in increased NAD^+^ levels to compensate the drop in NAD^+^ levels and enables Tregs to function in low glucose-high lactate environments. Specific factors in the atherosclerotic plaque microenvironment may downregulate Foxp3 expression and disrupt this metabolic advantage, leading to impaired suppressive Treg function and the progression of disease development.

The presence of low oxygen tension (hypoxia) has been detected in human and murine atherosclerotic plaques and is considered to be a crucial driver of atherogenesis [70]. Hypoxia-inducible factor 1α (HIF-1α) is the master transcription factor in orchestrating cellular responses to hypoxia. Under normoxic conditions, HIF-1α is continuously degraded via the ubiquitin-proteasome system, whereas hypoxia promotes its stabilization and transcriptional activity. Conflicting data have been reported concerning the effects of hypoxia and HIF-1α on Treg function [71]. Hypoxic conditions were found to promote the expression of Foxp3 in T cells [72,73]. Accordingly, these studies show that hypoxia increases Treg frequency in vitro and in vivo. Hypoxia-induced Foxp3 expression is antagonized following knockdown of HIF-1α. Conversely, stabilization or overexpression of HIF-1α strongly enhances Foxp3 expression, indicating that hypoxia upregulates Foxp3 via HIF-1α. In addition to increased Treg numbers, hypoxia-exposed Tregs also display stronger suppressive activity. Likewise, HIF-1α-deficient Tregs exhibit impaired suppressive function. Together, these results suggest that atherosclerotic plaque hypoxia may positively regulate Treg function. However, others have found opposite results and shown that hypoxia or HIF-1α itself induce the ubiquitination and subsequent proteasomal degradation of Foxp3 [74]. Moreover, T cell-specific deletion of HIF-1α increases Treg numbers in mice while reducing the abundance of Th17 cells [49,74]. Mechanistically, HIF-1α induces activation of the canonical Th17 transcription factor receptor retinoic acid receptor-related-orphan-receptor-gamma t (RORγt) [74]. Additionally, HIF-1α-induced upregulation of the glycolytic pathway serves as an important metabolic checkpoint for regulating the balance between Treg and Th17 differentiation [49]. Recently, it was shown that increased import of pyruvate into the mitochondria enhances the suppressive activity of HIF-1α-deficient Tregs under hypoxic conditions compared to wild type Tregs [75]. Hypoxia upregulates glycolysis by increased shunting of pyruvate to lactate, away from mitochondria. Deficiency of HIF-1α prevents this, resulting in enhanced mitochondrial metabolism to fuel the suppressive function of Tregs. Pharmacological inhibition of pyruvate transport into the mitochondria with UK5099 abolished the increased suppressive capacities of HIF-1α-deficient Tregs under hypoxia. Accordingly, treatment of Tregs with dichloroacetate to promote pyruvate entry into the mitochondria strengthens suppressive Treg function. Interestingly, while HIF-1α ablation in Tregs enhances suppressive activity, it decreases their migratory capacities. Again, these findings indicate that mitochondrial metabolism is tightly intertwined with the immunosuppressive functions of Tregs, and glycolysis is essential for their migration. Collectively, while opposing effects of hypoxia on Tregs have been described, the specific effects of hypoxia on Tregs in atherosclerotic plaques remain to be investigated.

**Figure 4:**
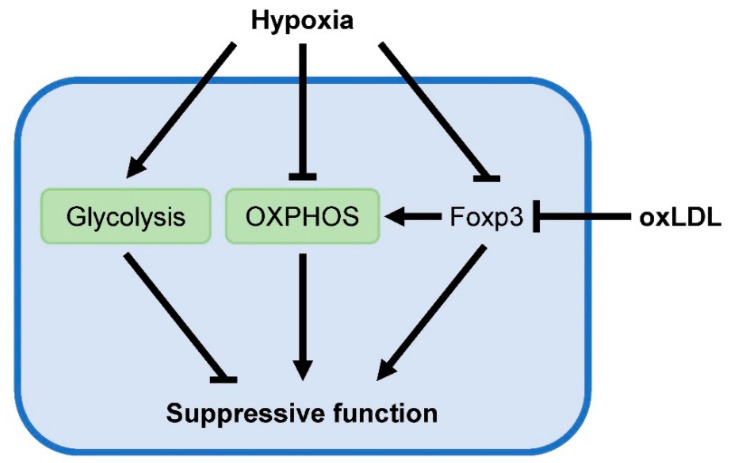
Putative influences of the atherosclerotic plaque microenvironment that negatively affect suppressive Treg function. Plaque hypoxia may induce proteasomal degradation of Foxp3 and reduce OXPHOS in Tregs, required for their suppressive functions. Additionally, oxidized LDL particles present in the plaque can downregulate Foxp3 expression and attenuate the suppressive Treg phenotype.

Oxidative modification of retained LDL particles in the arterial wall is considered to be a crucial driver of plaque formation during atherogenesis. Interestingly, incubation of human peripheral blood mononuclear cells or murine splenic CD4^+^ T cells with oxLDL reduces the amount of Tregs while keeping the effector T cell population largely unaffected, indicating that Tregs exhibit increased susceptibility to oxLDL [9,12]. Moreover, oxLDL was found to downregulate Foxp3 expression [9,67]. In line with this, oxLDL attenuates the suppressive capacities of human and murine Tregs [9,12]. Both apoptosis and reduced IL-2 signaling are proposed as underlying causes for the decreased Treg numbers following oxLDL treatment [12,67]. Additionally, intracellular accumulation upon oxLDL uptake may suppress the mevalonate pathway and impair Treg suppressive function, as detailed above. Altogether, these results clearly indicate that oxLDL negatively regulates Treg homeostasis. Although Foxp3-mediated metabolic adaptations allow Tregs to function at sites of low glucose and high lactate, unique environmental cues present within atherosclerotic plaques such as hypoxia and oxLDL may abrogate Foxp3 expression and impair the atheroprotective capabilities of Tregs (Figure 4).

## 9. Conclusions

Tregs are crucial modulators of immune responses and have been shown to be atheroprotective. Growing evidence indicates that Tregs exploit distinct intracellular metabolic pathways for their activities. While oxidative metabolism seems to be required for suppressive Treg function, glycolysis is closely associated with their proliferation and migration. Crucial drivers of atherosclerosis such as hypercholesterolemia and hypoxia may disturb Treg metabolism and drive disease progression. Nevertheless, the metabolic signature of Tregs during atherogenesis remains largely understood. Therefore, future research is warranted to identify novel metabolic targets that strengthen Treg-mediated atheroprotection and combat cardiovascular diseases.
